# The giant escape neurons of crayfish: Past discoveries and present opportunities

**DOI:** 10.3389/fphys.2022.1052354

**Published:** 2022-12-20

**Authors:** Jens Herberholz

**Affiliations:** Department of Psychology, University of Maryland, College Park, MD, United States

**Keywords:** crayfish, escape, neurons, circuits, synapses, history

## Abstract

Crayfish are equipped with two prominent neural circuits that control rapid, stereotyped escape behaviors. Central to these circuits are bilateral pairs of giant neurons that transverse the nervous system and generate escape tail-flips in opposite directions away from threatening stimuli.

## Introduction

Crayfish are equipped with two prominent neural circuits that control rapid, stereotyped escape behaviors. Central to these circuits are bilateral systems of giant neurons that transverse the nervous system and generate escape tail-flips in opposite directions away from threatening stimuli. This review describes the beginnings of crayfish giant circuit discovery and investigation, the fruitful advances that have been made over the past century, and it highlights some exciting opportunities for future research. After the giant escape neurons of crayfish were first anatomically identified in the late 19th century, they have been studied extensively by hundreds of scientists. That makes writing a comprehensive review a challenge, and it becomes unavoidable to omit some of the existing literature, unintentionally or not. There will be some personal bias in my account, and I apologize in advance to those whose undoubtedly important contributions to this field are not acknowledged, or not as much as they deserve. In addition, excellent reviews already exist as journal publications (e.g., Edwards et al., 1999) or in form of book chapters (e.g., Sillar et al., 2016), but a fresh look, including some of the more recent developments, might still be warranted. Important discoveries of broad significance have been made in the crayfish escape circuits. While the model might be considered less fashionable and even “non-traditional” in the context of current funding priorities, it has stimulated the curiosity of many research labs across different disciplines.

### General design of the nervous system and giant escape fiber pathways

**FIGURE 1:**
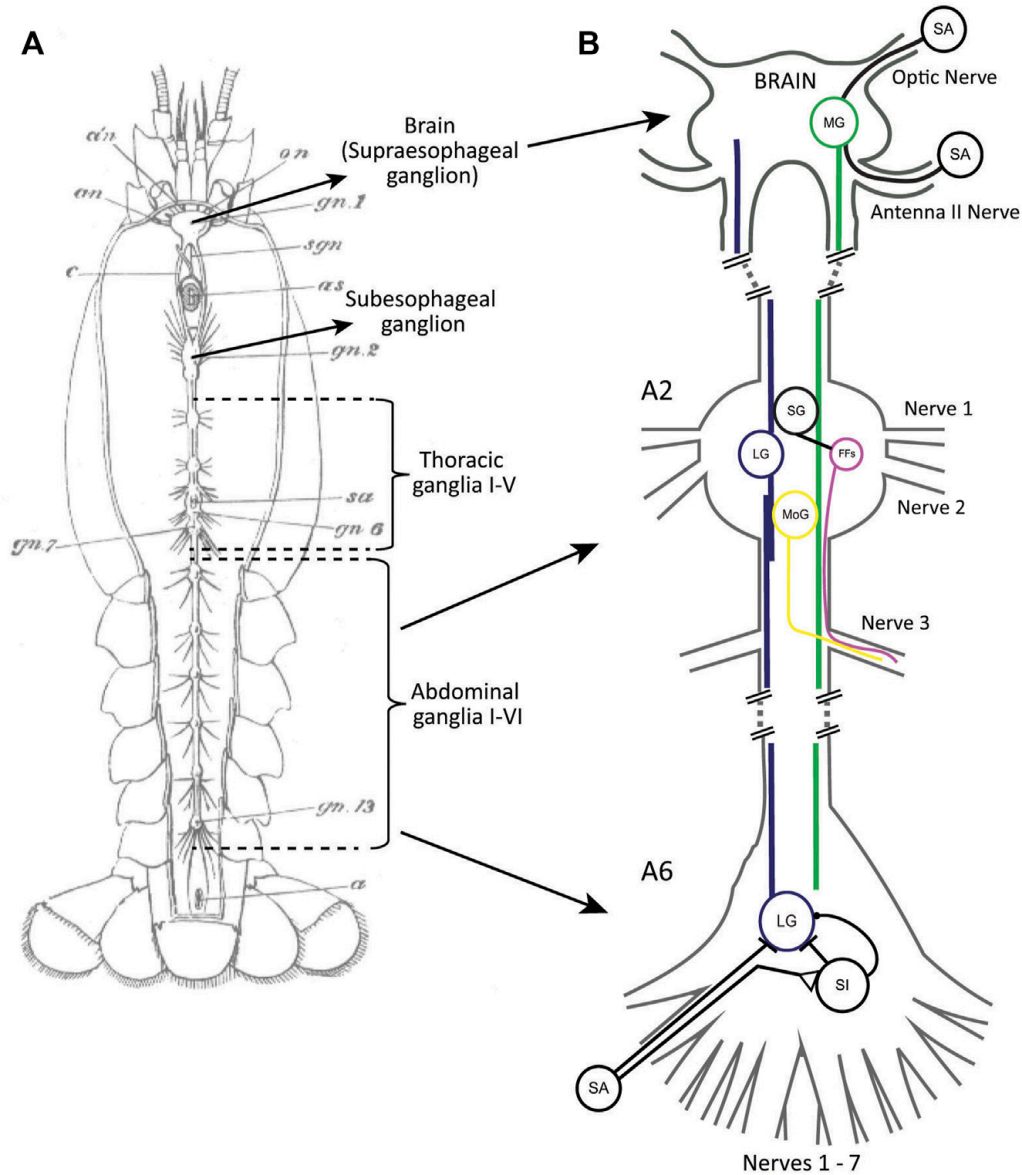
**(A)** A drawing of the decentralized nervous system of crayfish with major parts labeled. From Huxley (1880). **(B)** A simplified schematic of the giant neuron circuits with some of the input/output connections of MG (green) and LG (blue) indicated. MG receives sensory input of different modalities in the brain. LG receives mechanosensory input in the abdomen, both directly from primary sensory afferents (SA) and *via* sensory interneurons (SI). Some SIs also inhibit LG. The sensory pathway to LG is only shown for the last abdominal ganglion (A6). Both LG and MG activate the segmental giants (SG) as well as the motor giants (MoGs) in rostral abdominal ganglia (A2). The SGs activate groups of fast flexor muscles (FFs). Both the MoGs and FFs send axons to the muscles *via* the third ganglionic nerves. Only a single giant neuron (MG, LG, SG, MoG) is shown although these neurons exist as bilateral pairs in the ganglia. SAs, SIs, and FFs are groups of neurons. Axonal projections for MoGs and FFs are only shown for one nerve.

The basic organization of the crayfish nervous system is a decentralized structure that features a chain of ganglia localized in different body segments. From head to tail, this includes the supraesophageal ganglion (brain), the subesophageal ganglion, five thoracic ganglia, and six abdominal ganglia (Figure 1A). The bilateral pairs of giant escape neurons (lateral and medial) receive their main sensory inputs on opposite sides of the body. The medial giant (MG) neuron is excited by sensory input to the brain while the lateral giant (LG) neuron is primarily stimulated by caudal mechanosensory inputs. Both giant fibers activate a discrete set of postsynaptic premotor (i.e., segmental giants) and motor (i.e., motor giants) neurons in the thoracic and abdominal ganglia (Figure 1B). This leads to different patterns of muscle activity that produce rapid tail flexions in all abdominal segments (MG) or around the thoracic-abdominal joint (LG), resulting in escape trajectories away from the point of stimulation.

### The beginnings

George Edwin Johnson is often credited with the discovery of the crayfish giant fibers almost 100 years ago (Johnson, 1924), but he refers to earlier work by Krieger (1880) who identified large medial and lateral fibers in cross-section of the nerve cord. In addition, Retzius (1890) used methylene blue staining to show medial giant fibers in thoracic and abdominal ganglia. Johnson, however, was the first one to provide a detailed anatomical description of the giant fibers combining cross-sectioning and dye staining. He reported that the medial giant fibers (MGs) originate in the brain where their cell bodies are located, and they project to the last abdominal ganglion. He also described the lateral giant fibers (LGs), which he named *segmental* giant fibers because he recognized that they are single neurons tightly coupled to their posterior/anterior homologues in all abdominal and the most caudal thoracic ganglia. Johnson also found what appeared to be intraganglionic coupling between the bilateral pair of LGs, and he was able to identify connections between the giant fibers and the giant motor fibers (MoGs). He further traced the axons of the MoGs in the third segmental nerves and proposed a putative motor pathway to the trunk muscles and related to tail flexion (Figure 1B). Lastly, Johnson discovered that the last abdominal ganglion contains two sets of LGs and MoGs, as well as twice as many nerves compared to anterior ganglia, which he attributed to the nature of an additional, fused terminal segment. Given that neuron-to-neuron communication was thought to happen *via* (chemical) synapses or by transmission *via* a nerve-net structure at the time, Johnson recognized the “strikingly unique” connection that the LGs make with their segmental homologues. In hindsight, this is not surprising because electrical neurotransmission was unknown at the time, and it took another 30 years before the first clear evidence for an electrical synapse was provided by Furshpan and Potter (1957). Interestingly, it was in the same circuit, between the LGs and MoGs, where this important discovery was made. Several years later, this led to the identification of “septate junctions” that connect the LGs in each segment along the nerve cord (Watanabe and Grundfest, 1961).

Building on Johnson’s comprehensive anatomical studies, C.A.G. Wiersma performed the first functional investigation of the crayfish giant circuits (Wiersma, 1938). In a brief paper published in *Proceedings of the Society for Experimental Biology and Medicine*, he reported that a single stimulus to one of the giant fibers “causes a twitch-like contraction of the flexor muscles of the tail”. Wiersma partially dissected the animal and used extracellular electrodes to stimulate each of the four giant fibers he individually separated from surrounding axons and tissue in the desheathed brain connectives (“oesophageal commissures”). He found that a suprathreshold stimulus to either giant fiber would cause the tail flexion, an all-or-nothing response. Cutting of the third nerve roots of abdominal ganglia (which contain the axons of the MoGs; Figure 1B) prevented the tail-flip after giant fiber stimulation, suggesting that all giant neurons utilize the same motor pathway.

Wiersma’s ability to perform such clever experiments on individual neurons was undoubtedly aided by the large size of the giant fiber axons, which are between 100 µm (LGs) and 200 µm (MGs) in diameter in the brain connectives of adult crayfish and visible under a standard light microscope, although their diameters vary widely in animals of different size (Wiersma 1947; Wine and Krasne 1972; Glantz and Viancour 1983; Edwards et al., 1994a).

**FIGURE 2:**
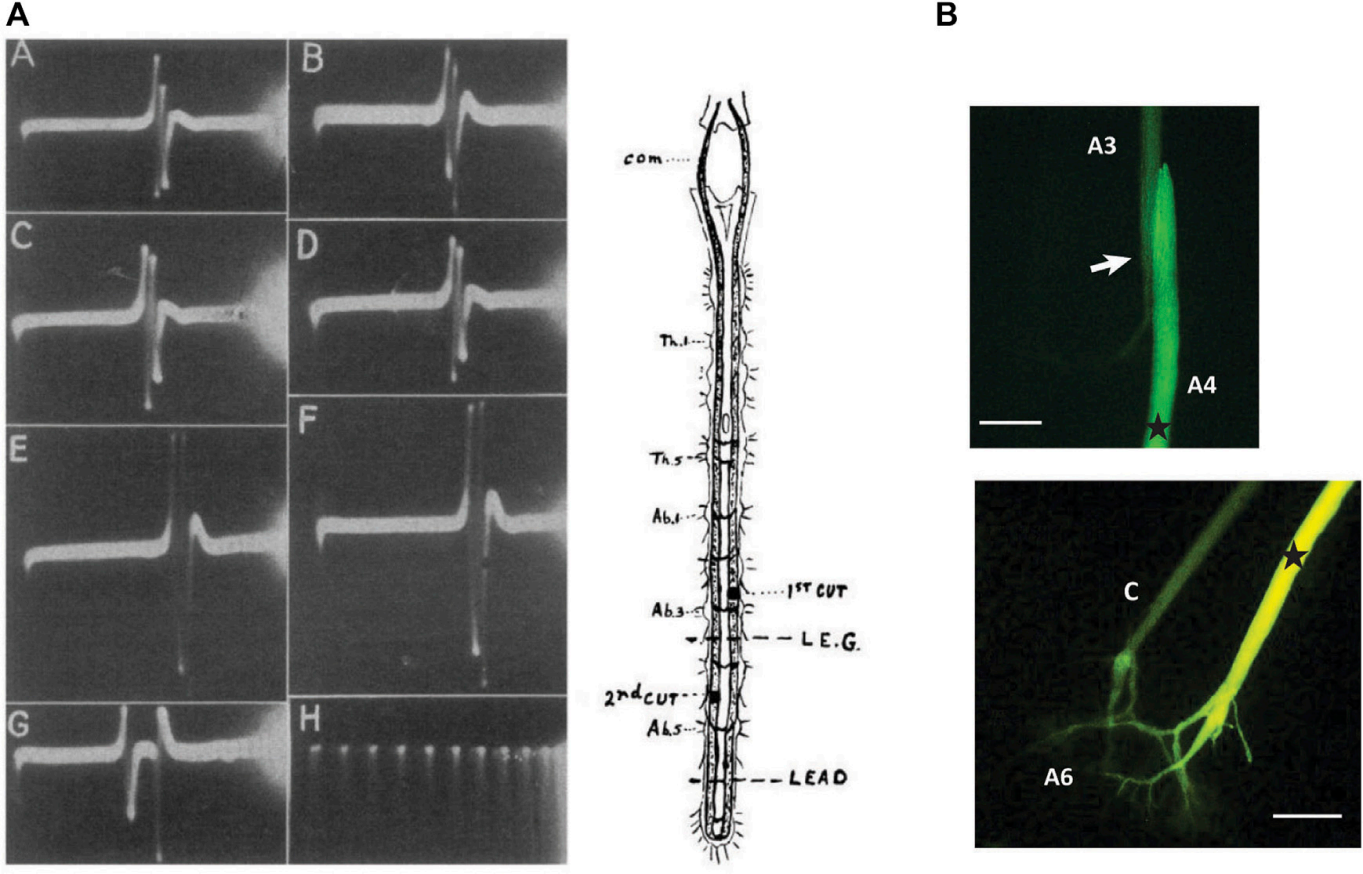
**(A)**. Demonstration of the functional connections between bilateral LG neurons. After stimulating one LG in one of the brain connectives (com), two LG spikes can be recorded between the fifth and sixth ganglia of the abdominal nerve cord. Top traces **(A,B)** are after stimulation of left and right LG, respectively. Traces C-F are recordings after cut of the right LG (first cut) and cut of the left LG (second cut). Bottom traces, (G,H) are recordings between A3 and A4 and time in milliseconds. *From* Wiersma (1947) (Figure 1). (Reused with permission Copyright Journal of Neurophysiology). **(B)** Injection of fluorescent dye (Lucifer Yellow) into the LG neuron in the fourth abdominal ganglion of the crayfish nervous system shows dye labeling of the adjacent LG neuron in the next, more anterior, segment and highlights the electrical synapses (“septate junctions”) that connect the segmental LG neurons. **(B)** Injection of the same dye into one LG neuron (right side) in the sixth abdominal ganglion illustrates the electrical coupling between the pair of LG neurons with dye spreading into the contralateral LG (C). Stars indicate approximate injections site. Arrow indicates the septate junction. Scales = 400 µM (based on axon diameter). (Credit: Jens Herberholz; the images are for illustrative purposes only and do not introduce new knowledge).

In 1947, Wiersma published a paper that further detailed the relationship between giant fiber activation and tail-flip behavior. He reported that a superthreshold stimulus to either MG was sufficient to generate a stereotyped escape tail-flip. Using recording electrodes placed on the nerve cord in caudal locations, he first reported a conduction velocity of 15–20 m/s for the MG neurons. However, when one LG fiber was stimulated in the brain connectives, Wiersma observed two LG spikes in the cord, confirming the functional coupling between both LGs that Johnson had proposed years earlier (Figure 2A). In addition, after cutting one of the LGs between the stimulating and recording electrode, Wiersma demonstrated a conduction velocity of 10–15 m/s for an ipsilateral LG spike and proposed that crossover of action potentials between one LG and its contralateral homologue is restricted to the posterior thoracic ganglia and the first five abdominal ganglia. Wiersma further suggested a rostral receptive field for the MG, which is primarily activated by optical stimulation, whereas the LG’s mechanoreceptive field was thought to be restricted to the posterior thorax and abdomen. In this seminal paper, Wiersma also recorded action potentials in the nerves that originated from the third abdominal ganglion after stimulation of the giant fibers in the brain connectives. His results confirmed that both LG and MG will produce a single, large spike in the third nerve (i.e., the MoG axon connecting to the abdominal flexor muscles), while no activity was observed in the second nerve, and some, but less reliable activity was seen in the first nerve.

Wiersma’s contribution to the scientific community cannot be overstated. Taking advantage of the crayfish as a model system with large, accessible, and individually identifiable neurons that link directly to behavior, Wiersma laid the groundwork for future generations of researchers who realized the great potential of the crayfish giant circuits for investigating important scientific questions that ranged from molecular biology to robotics.

20 years later, Stretton and Kravitz (1968) introduced the first fluorescent intracellular dye (Procion Yellow), and this discovery paved the way for injections into single neurons, including the LGs and MGs as well as other parts of the giant circuits. Because intracellular dyes of low molecular weight will pass through gap junctions, the technique has been successfully used to show the coupling of the LGs to their contralateral homologue and across the septate junctions. An example is shown in Figure 2B.

### Behavioral relevance of the giant fiber systems

In 1972, Jeff Wine and Frank Krasne published a paper that beautifully illustrated the adaptive behavioral value of parallel giant escape circuits in crayfish. By implanting recording electrodes into the abdomen of freely behaving animals, they were able to measure giant fiber activity in response to various “natural” stimuli while observing the resulting behavioral actions. Based on prior results showing that crayfish can tail-flip *without* giant fiber activation (Schrameck, 1970), and discrete differences that exist in motor patterns and behavior between LG and MG tail-flips (Larimer et al., 1971), they tapped, pinched, or touched different body parts and analyzed the corresponding neurobehavioral outcomes. They found that strong, phasic taps to the abdomen stimulated the LG neurons and propelled the animal upward and forward, away from the point of stimulation. Taps to the most rostral body parts, however, evoked MG-mediated tail-flips that propelled the animals backwards. Visual stimuli were much less successful in activating MG tail-flips but were often seen to produce tail-flips without giant fiber activation. Both tail-flips produced by giant fibers had very short latencies (∼4 ms) whereas non-giant tail-flips were initiated with much longer (∼200 ms) and more variable delay. Non-giant tail-flips were most successfully elicited with gradual stimuli, and contrary to giant fiber mediated tail-flips, which were highly stereotyped, they differed in escape direction and angle. Moreover, non-giant tail-flips were abolished when the nerve cord was cut above the thorax, suggesting control of non-giant tail-flips in the subesophageal ganglion. The notion that non-giant tail-flips are controlled by the subesophageal ganglion was later revised by Lee et al. (1995) who demonstrated persistent activity of non-giant motor neurons in abdominal segments after thoracic-abdominal nerve cord transection.

**FIGURE 3:**
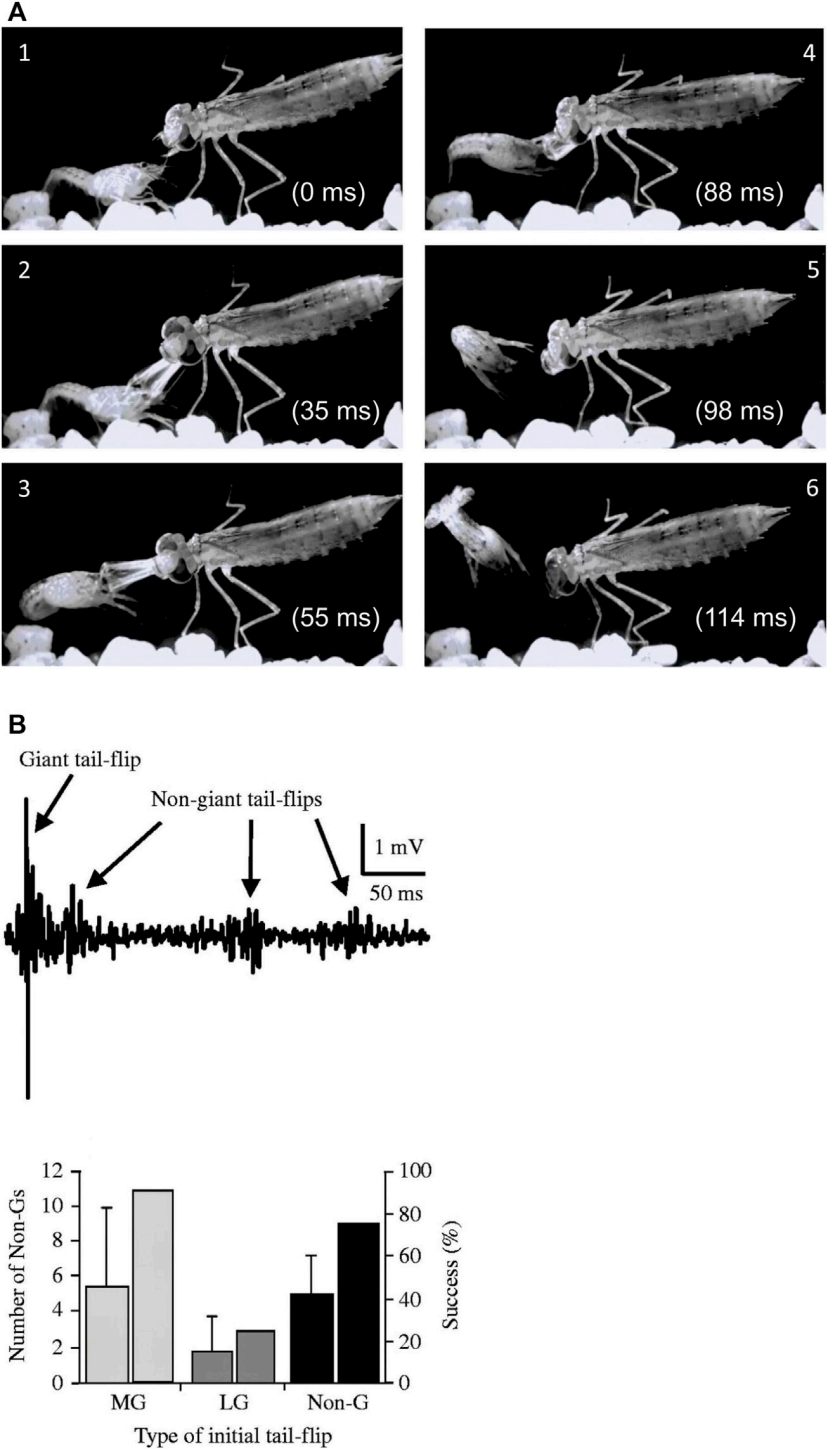
**(A)**. Single video frames (1–6) from a staged encounter between a dragonfly nymph and a juvenile crayfish recorded at 1,000 frames/sec. The crayfish responds to the frontal attack with an instantaneous backward tail-flip but is still captured by the nymph’s mandibles. It quickly employs a second, sideways-directed tail-flip to successfully free itself. (Credit: Jens Herberholz; the images are for illustrative purposes only and do not introduce new knowledge)*.* **(B)**. Top: Bath electrode recordings of field potentials showing the initial giant mediated tail-flip and three subsequent non-giant tail-flips produced by a juvenile crayfish in response to a dragonfly nymph attack. Bottom: Initial MG and non-giant tail-flips are regularly followed by non-giant tail-flips after capture and improve the overall escape success to ∼80%. The numbers are lower if the initial, unsuccessful response to the attack was an LG tail-flip, presumably due to the capture point near the tail, which impeded subsequent non-giant tail-flips. From Herberholz et al. (2004) (Figure 7). (Reused with permission from the Company of Biologists).

The *survival value* of the escape circuits was tested many years later when freely behaving juvenile crayfish were paired with a natural predator (Herberholz et al., 2004). An example of such an encounter between a juvenile crayfish and a dragonfly nymph is shown in Figure 3A. The activity of the different escape circuits in response to predator attacks was recorded with a pair of electrodes fixed to the sides of a small aquarium, a technique that was previously developed to measure escape circuit activation in crayfish during intraspecific fights (Herberholz et al., 2001). Because each tail-flip circuit creates a signature field potential in the water surrounding the animal, this method provided the unique advantage of non-invasive neural recordings. When juvenile crayfish were attacked by dragonfly nymphs, they engaged all three escape circuits albeit at different success rates. Since most attacks were directed to the front, MG mediated tail-flips were most common. Attacks to the rear evoked LG tail-flips whereas non-giant tail-flips resulted from attacks to the middle parts of the crayfish body. The escape rate was quite high (44–50%) if the attack triggered a giant-mediated tail-flip, but much less for non-giant tail-flips (20%). The poor escape performance for non-giant tail-flips is likely attributed to their longer response latencies, which were twice the duration of giant fiber mediated tail-flips. However, the average escape latencies for non-giant tail-flips were still significantly faster in response to the predator attack when compared to a mechanical tap using a handheld probe, while this was similar for MG and LG tail-flips. This suggested that crayfish monitor the approaching predator to prepare for a possible attack, thus “priming” the non-giant system for faster responses to anticipated attacks. In addition, crayfish that were captured by the nymphs often generated a series of non-giant tail-flips, especially if the attack first triggered an unsuccessful MG- or non-giant tail-flip, which sometimes freed them from the predator’s mandibles and improved their overall escape success to ∼80% (Figure 3B).

Together, these studies illustrated the behavioral importance of the three escape circuits in crayfish. The two giant fiber circuits allow the animals to quickly escape strong and phasic danger signals directed to the front or rear, and the unique activation patterns of abdominal muscles lead to appropriate motor action. Although highly efficient, the giant-mediated responses lack behavioral plasticity. MG and LG circuits are built for speed and the behavior is stereotyped and “involuntary”. If time permits, the non-giant circuit comes into play, which adds control over the direction and angle of escape. When in the fangs of an insect larva (and probably other predators as well), both versions can be combined to maximize escape success.

Although comparison to other taxa is outside the scope of this review, it is notable that the nature of “fast and slow” escape circuits is a fairly universal phenomenon. Examples include the giant Mauthner cells and parallel circuitry in teleost fish that mediate short- and long-latency escapes as well as the giant fibers and non-giant pathways in *Drosophila* (and other insects) that underlie similar behavioral functions (e.g., Burgess and Granato 2007; Card 2012).

### Mapping of the giant fiber circuits

Using the LG circuit, Furshpan and Potter (1957), Furshpan and Potter (1959) identified the first electrical synapse in nervous systems. Since then, the LG circuit has been almost entirely mapped out, from the motion-sensitive hairs that cover the crayfish tail to the motor neurons that activate the abdominal muscles, and everything in-between (Figure 1B). Most neurons and synapses in the circuit have been described, including a complex system of inhibition.

#### The LG circuit

There has been a notable imbalance though in both research efforts and resulting knowledge across the three escape circuits. The LG circuit is arguably one of the best understood neural circuits in the animal kingdom, whereas much is still to learn about the MG circuit, and little is known about the non-giant circuit (Edwards et al., 1999). This is mainly based on practical considerations. It is easier to trace out to the periphery from large giant neurons that provide accessible starting points. Moreover, compared to the MG, the LG has the advantage of being a local circuit that repeats in each of the abdominal ganglia. The ventral nerve cord with all abdominal ganglia can be removed from the animal and survives for many hours when bathed in saline solution; this *ex vivo* preparation is well suited to study all circuit features without the need of keeping the more fragile brain (with the MG neurons) alive.

The LG neuron is activated by tactile or hydrodynamic forces applied to the tail or abdomen. Hair mechanoreceptors, which are directionally sensitive, activate primary afferents that project into the abdominal ganglia (Wiese 1976; Wiese et al., 1976). The afferents project centrally through sensory nerves and their receptive fields are, at least partially, maintained on the level of the interneurons (Zucker et al., 1971; Calabrese 1976; Antonsen and Edwards 2003). In the last abdominal ganglion, LG also receives monosynaptic excitatory input from proprioceptive afferents that monitor movements of the tailfan (Newland et al., 1997; Newland et al., 2000).

The afferents form direct electrical synapses with the LG neuron, and they make chemical synapses with a number of interneurons. The interneurons connect to LG *via* mixed electrical and chemical, including inhibitory, synapses (Lee and Krasne 1993). The excitatory inputs to LG are thus biphasic, an early electrical excitatory postsynaptic potential (EPSP), the α-component, is followed with short delay by a second chemically mediated EPSP, the β-component (Krasne, 1969; Zucker, 1972a). The β-component of the EPSP is followed by postexcitatory inhibition (Vu et al., 1997), presumably produced by GABAergic interneurons, and a long-lasting, variable γ-component, which present a mixture of excitatory and inhibitory signals (Berkowitz et al., 1998). In juvenile crayfish, the α-component of the LG EPSP is large enough to reach threshold and trigger an action potential, but in adult animals, it is much more attenuated due to isometric growth of the LG, and the spike always rises from the depression-prone β-component (Edwards et al., 1994b).

**FIGURE 4:**
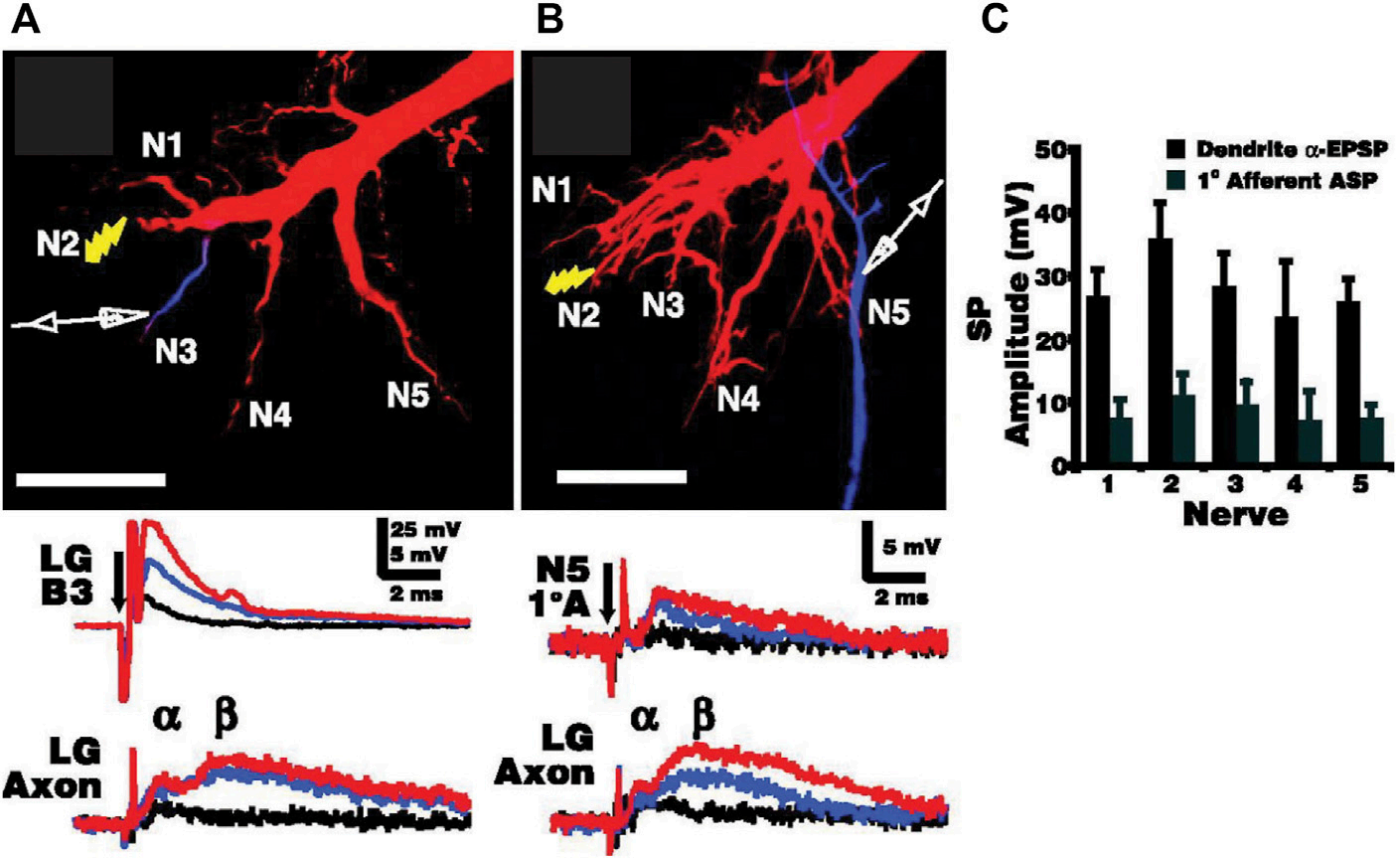
Electrotonic coupling between primary afferents across the sensory input pathways to the LG. **(A)**. Increasing electrical stimulus (shock) intensity applied to sensory nerve 2 (N2) in the last abdominal ganglion (A6) leads to postsynaptic potentials (PSPs) in the LG dendrite (LGB3) of increasing sizes (black, blue, and red traces) as well as smaller, increasing biphasic PSPs in the initial axon segment (LG Axon). α and β refer to the electrical and chemical components of the LG PSP, respectively. **(B)**. The same stimulus sequence of increasing intensity also elicits small PSPs in spatially removed primary afferents (1°A) such as those projecting to the LG dendrites *via* N5, indicating spread of LG potentials across the afferent network. LG was injected with Texas Red, and the location of the recording electrode was confirmed with Cascade Blue injections. Scale bars: 100 µm. **(C)**. Averaged amplitudes of synaptic potentials (with standard deviation) recorded in nerves N1-N5 for both the dendritic α-component of the LG EPSP and for the antidromic synaptic potentials (APS) recorded in primary afferents (*N* = 6). Afferents closer to LG dendritic inputs receive larger antidromic potentials. From Antonsen et al. (2005) (Figure 3). (Reused with permission. Copyright (2005) Society of Neuroscience.)

Primary afferents that couple electrically to the LG neurons are also connected to each other by non-rectifying gap junctions. This allows local depolarization to spread into neighboring afferents and creates a lateral excitatory network that enables rapid amplification of local sensory inputs to LG (Herberholz et al., 2002; Antonsen et al., 2005). Afferents in different sensory nerves also couple to each other through the LG dendrites as synaptic potentials in LG spread antidromically into other unstimulated afferents, which further contributes to afferent recruitment and lowers the threshold for slightly delayed stimuli (Figure 4). The LG itself acts as a coincidence detector, and is maximally stimulated by simultaneous sensory inputs, which makes it most sensitive to phasic stimuli (Edwards et al., 1998).

The afferent-interneuron chemical synapse is nicotinic cholinergic (Miller et al., 1992) and depresses after repeated stimulation (Zucker, 1972b). This was an exciting finding at the time because it was the first to uncover a specific neurocellular mechanism underlying behavioral habituation. The concept was later revised, however, as it became apparent that behavioral habituation is also mediated by descending tonic inhibition from the brain to the local LG circuits (Krasne and Teshiba, 1995; Shirinyan et al., 2006).

Once the LG fires an action potential, it activates the MoGs in the anterior abdominal ganglia (A1-A3), and the posterior thoracic ganglia (T4-T5), which causes the jack-knife tail-flip that pitches the animal upward and forward (Heitler and Fraser 1993). However, the LG activates several other postsynaptic neurons as well. Electrical coupling to the segmental giant (SG) neurons engages a pathway that amplifies the output to the fast-flexor motor neurons (Kramer et al., 1981a; Roberts et al., 1982; Heitler et al., 1985). The LG spike also causes depolarizing inhibition of the primary afferents *via* the SG neuron. This primary afferent depolarization (PAD) prevents habituation of the afferent-interneuron chemical synapse during the tail-flip (Kennedy et al., 1974; Kirk 1985).

The SG drives both primary afferent depolarizing interneurons (PADIs) as well as corollary discharge interneurons (CDIs) in the rostral ganglia of the abdominal nerve cord. The CDIs, of which only two have been individually identified (CDI2, CDI3) excite the flexor inhibitor after LG or MG activation, and they participate in inhibition at the afferent-interneuron synapse (Wine and Mistick 1977; Kramer et al., 1981b).

The LG also inhibits itself. The LG action potential is immediately followed by a depolarizing inhibitory PSP (dIPSP), which lasts for tens of milliseconds, rendering the LG unresponsive to any sensory stimulation for the duration of the tail-flip (Roberts, 1968). This “recurrent” inhibition prevents the LG from being activated by the behavior it produces, which evokes massive stimulation to its sensory input pathways. The inhibition is depolarizing due to the opening of chloride channels and efflux of chloride ions, which are highly concentrated in the LG neuron relative to the outside. The depolarization is inhibitory because the reversal potential of chloride is lower than LG threshold, and the sustained depolarization mainly causes inactivation of sodium channels (Edwards et al., 1991). Recurrent inhibition is located near the LG spike initiation zone, and it is absolute, meaning no excitation can overcome it (Vu and Krasne 1992). The inhibitory neurons responsible for it have not been identified, but its minimal delay after the action potential makes a polysynaptic pathway unlikely. Although the LG dIPSP can be suppressed with picrotoxin, a chloride channel blocker, it has not been established whether this is based on antagonism of GABA-gated or glutamate-gated chloride channels, both of which exist in crayfish (Heitler et al., 2001; Nagayama 2005).

Despite a near complete picture of the LG circuit, additional discoveries have been reported in recent years. Although the receptive field of the LG was long considered to be limited to the rear of the animal, the LG does receive subthreshold excitation from rostral sensory areas (Liu and Herberholz, 2010). For example, electrical stimulation of the antenna II nerve or protocerebral tract (i.e., optic nerve) caused small EPSPs in LG, and when those inputs were combined with subthreshold stimulation of tail afferents, they brought LG to threshold. The small LG EPSPs are caused by monosynaptic inputs from identified descending interneurons that receive sensory signals in the brain. This adds an interesting new component to the LG circuit, indicating that potential danger signals that are perceived with rostral sensory systems might lower the LG threshold to caudal attacks and thereby contribute to behavioral escape decisions.

**FIGURE 5:**
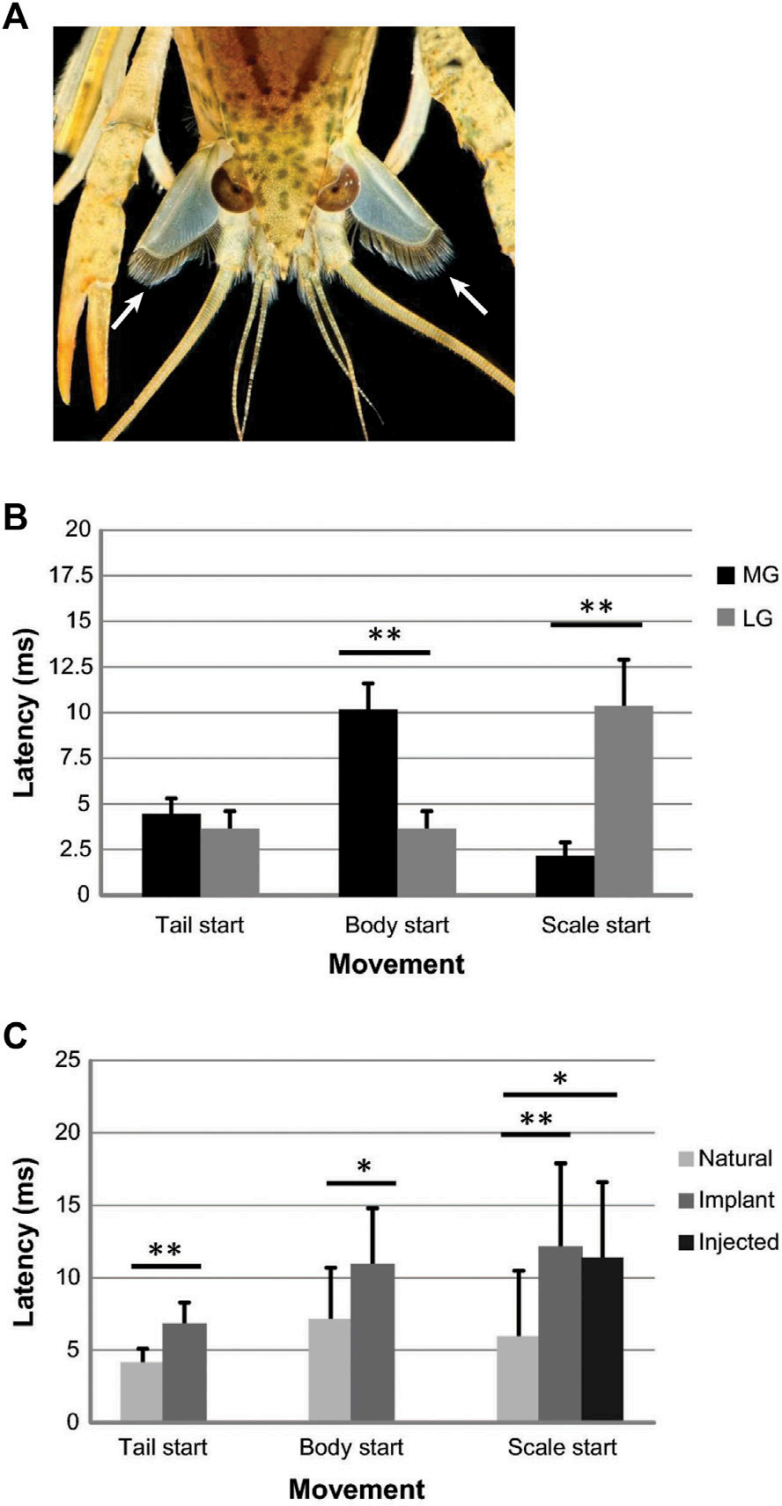
**(A)**. Photograph of a juvenile crayfish during an LG-mediated tail-flip. Frontal view shows the extended antennal scales below the eyes that are fully extended at the time of tail flexion (arrows). The function of the hairs that line the inner ridges of the scales is currently unknown. (Credit: David D. Yager & Jens Herberholz, University pf Maryland. The image is for illustrative purposes only and does not introduce new knowledge). **(B)**. Tactile (“natural”) stimulation of MG or LG tail-flips with pokes to the head or tail, respectively, reveal differences in the onset times of scale extensions compared to other body movements. For MG tail-flips, the scales extend before the tail and body start to move, whereas scale extensions are delayed during LG tail-flips and start after the tail and body moves. **(C)**. Comparison of latencies to move the tail, body, and scales after LG and MG tail-flips (data pooled). Giant-mediated tail-flips were evoked with natural stimulation (*N* = 15), implanted electrodes around the brain connectives for stimulating MG tail-flips and around the ventral abdominal nerve cord for stimulating LG tail-flip (*N* = 12), or intracellular current injections into the giant neurons (*N* = 6). Natural stimuli produced significantly shorter latencies for movement onsets, including scale extensions in restrained animals. From Herberholz et al. (2019) (Figures 2, 6). (Reused with permission, Copyright 2019; Springer-Verlag GmbH Germany).

LG motor output was also long considered to be locally restricted, primarily to the abdomen and caudal part of the thorax. However, a recent study found that the LG also activates a set of head appendages (i.e., antennal scales; Figure 5A), and it does this with superb temporal precision (Herberholz et al., 2019). LG-mediated scale extensions are delayed compared to body movements and full extension of the scales coincides with full flexion of the tail, possibly aiding in upward thrust. Scale extensions are also triggered during MG tail-flips but start much quicker after stimulation of the MG neurons (Figure 5B) and may thus act as rudders and/or help with avoiding rotation during backward motion. Interestingly, when movements of the tail, body, and scales were measured with natural stimuli, implanted electrodes, or intracellular current injections into the giant fibers, the delay was always significantly shorter for natural stimuli (i.e., pokes with a handheld probe), indicating that the giant circuits might be primed under natural conditions (Figure 5C). As mentioned earlier, a similar effect has been reported for non-giant tail-flips (Herberholz et al., 2004).

#### The MG circuit

The MG circuit has been much less explored, especially its sensory input pathways, but relevant information exists (Figure 1B). Wiersma (1947) proposed that the soma of both MGs are located in the brain, and the two MGs can excite each other. Horiuch et al. (1966) and Horiuchi et al. (1971) confirmed the anatomical organization with stains of serial brain sections and provided first descriptions of MG branching patterns, including innervation of antenna I/II. Raymon Glantz and colleagues provided detailed neuroanatomical and physiological description of the MG neurons. Using intracellular dye injections, they confirmed electrical coupling between two MG cells in the brain (Glantz and Kirk 1980), confirmed branching into olfactory and antennal lobes, and, using electrophysiological techniques, described basic neuronal parameters such as MG time/length constants, membrane resistance and membrane capacitance (Glantz and Viancour, 1983). Mellon and Christison-Lagay (2008) demonstrated that electrical stimulation of the bases of the antennules (i.e., antenna I) evoked action potentials in crayfish MG neurons, and the authors suggested that these inputs are mediated by mechanosensory hairs located on the flagella of the antennules. Similar results have been obtained after electrical stimulation of the antenna II nerve, which strongly depolarized the MG neurons and occasionally caused action potentials (Liu and Herberholz 2010; Swierzbinski and Herberholz 2018).

Behaviorally, MG tail-flips can be evoked with phasic tactile stimuli directed to the antenna I/II, rostrum, claws, and legs, as well as a rapidly approaching visual object, although visual activation was initially considered to be much less effective (Wine and Krasne 1972). However, the susceptibility of the MG neurons to visual inputs was later demonstrated by recording MG action potentials in freely behaving (and foraging) juvenile crayfish exposed to rapidly approaching shadows (Liden and Herberholz 2008; see below). In addition, modulation of MG threshold was also suggested to include olfactory signals, providing further evidence that the MG circuit integrates multiple sensory modalities (Liden et al., 2010; Schadegg and Herberholz 2017).

Similar to the LG neurons, the motor output of the MGs also extends beyond the abdomen and thorax. The MGs drive the extension of the aforementioned antennal scales, but they do this with much shorter temporal delay (Herberholz et al., 2019; Figure 5B). When the MG neurons were activated in freely behaving animals by poking the head with a handheld rod, full extension of the scales was completed *before* the animal began to move backward. For LG tail-flips, however, the extension was precisely timed to coincide with full flexion of the tail. This suggested that the scales have different functions, possibly acting as a rudder for steering in MG tail-flips while increasing the “squeeze force” of water between scales and tail-fan for LG tail-flips. However, relevant behavioral investigation to test this hypothesis is still missing.

As we have seen, much is still unknown about the MG circuit, and not surprisingly, this is a consequence of increased complexity and reduced accessibility. Yet, the multimodal sensory nature of the MG circuit offers great promise for uncovering neurobehavioral mechanisms underlying multisensory integration.

The cellular interactions between the two giant fiber systems have also been sparsely studied. Roberts (1968) demonstrated that MG action potentials generated a long-lasting depolarizing inhibition in LG neurons. This inhibition, like LG’s recurrent inhibition, was sensitive to picrotoxin, and prevented LG activation for the duration of an MG tail-flip. This makes good sense because LG activation during an MG tail-flip should be considered maladaptive. For example, a frontal attack that drives the crayfish backwards, possible into an obstacle, which then activates the LG circuit, would immediately propel the animal forward into the predator or opponent that evoked the initial response. The inhibitory interneurons, most likely GABAergic, that mediate the fast inhibition of LG have not been identified. Surprisingly, whether this MG-LG inhibitory relationship exists in the opposite direction is currently unknown and needs further investigation.

### Changing the threshold of the giant escape neurons

#### Behavioral modulation

One of the great benefits of studying the giant fiber circuits in crayfish is their unambiguous link to concrete behavioral outcomes. If the giants fire, the animal will tail-flip. Thus, it can be safely assumed that changing the *threshold* of giant fiber activation provides crayfish with much needed flexibility in varying environments and situations. One such mechanism is tonic inhibition. This form of inhibition is commonly found in the nervous systems of many species and is distinguished from synaptic inhibition by its global release patterns, slower activity, and the binding to specific receptor subtypes, often extrasynaptically, and mostly of the GABAergic type (Farrant and Nusser 2005). Most of the work on crayfish tonic inhibition (TI) has been provided by Frank Krasne and colleagues. TI in crayfish descends from the brain and changes the excitability of the LG neurons locally in the abdomen. This was demonstrated by cutting the brain connectives, which eliminated TI (Vu and Krasne 1993). TI is picrotoxin-sensitive, presumably acts on inhibitory receptors that are found distally on the LG dendrites, and it can be overcome by strong excitatory inputs, making it “relative” rather than “absolute” such as LG recurrent inhibition (Lee and Krasne 1993; Vu et al., 1993). As mentioned earlier, TI also contributes to habituation of the LG circuit (Krasne and Teshiba 1995).

Most importantly, TI allows the crayfish to discretely up- or downregulate giant fiber excitability by increasing or decreasing shunting of excitatory inputs to the dendrites (e.g., Vu et al., 1993), and thus allows to modulate escape behavior in various contexts. For example, when crayfish were physically restrained, the thresholds for LG and MG tail-flips increased, but these threshold changes disappeared when the brain connectives were cut, suggesting TI as the underlying mechanism (Krasne and Wine 1975). The purpose of rendering the giant neurons less responsive during restraint is hypothesized to be based on the many elements that are shared between giant- and non-giant circuits, including sensory input pathways, which allows for a more voluntary, directional type of escape. This idea is further supported by the aforementioned finding that juvenile crayfish exclusively engaged non-giant circuitry after they had been firmly captured by a dragonfly nymph, while the initial response to an attack was predominately giant-mediated tail-flip escape (Herberholz et al., 2004).

Threshold modulation of the LG neurons was also illustrated when crayfish were offered food of different sizes (Bellman and Krasne 1983). LG activation threshold was reduced when crayfish ate food, but only when the size of the food prevented the animals to hold on to it when tail-flipping. If the food item was small, LG threshold did not change (or went up), because the animals did not have to sacrifice the tasty meal for the equally important response to a predator. The site of action is the LG neuron itself (and presumably mediated by TI), which responded less - or more - to dendritic inputs from primary afferents and sensory interneurons (Krasne and Lee 1988).

**FIGURE 6:**
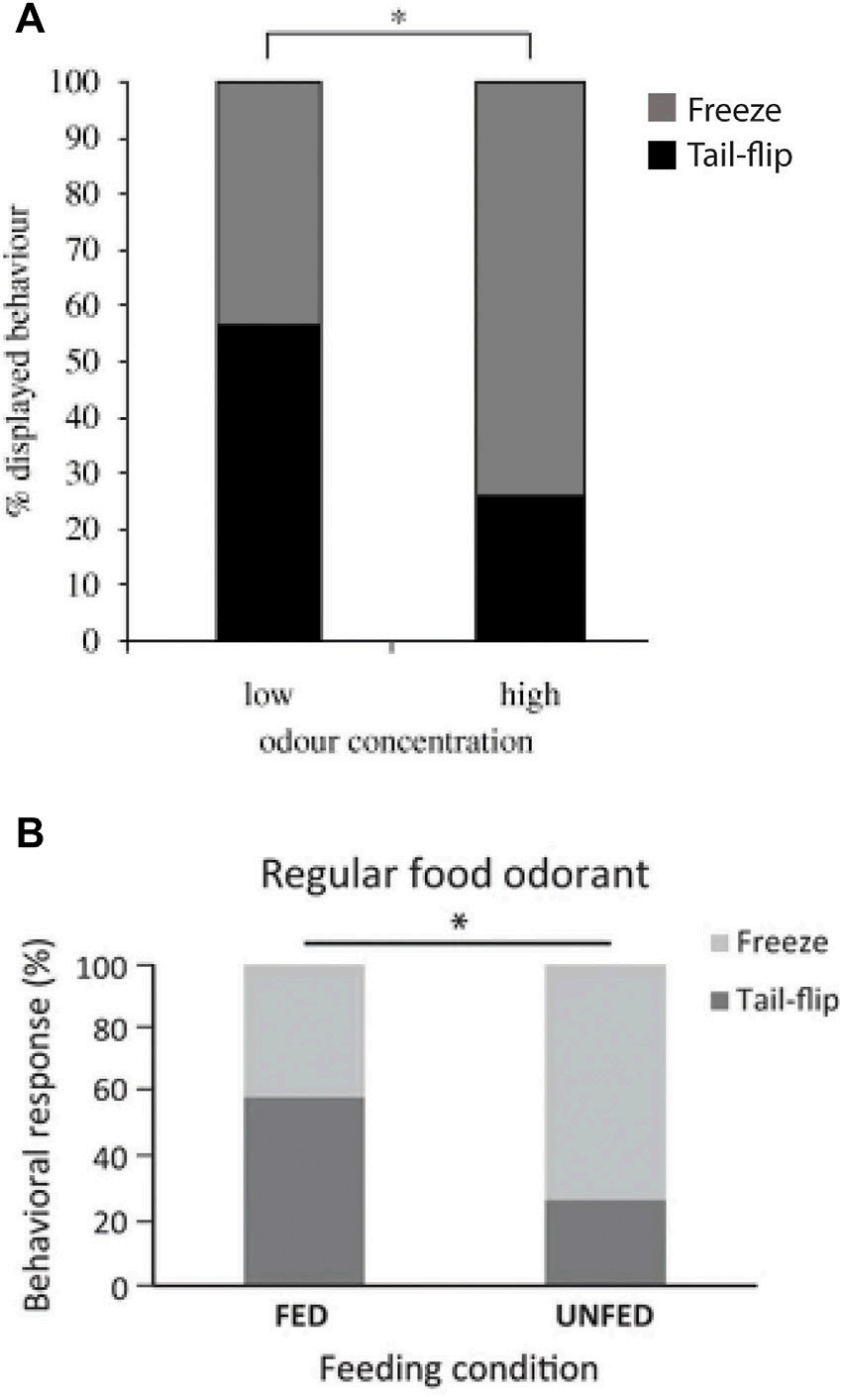
**(A)**. In response to an approaching visual danger stimulus, foraging crayfish (*N* = 61) freeze more often if the expected food quality is high and tail-flip more often if it is low. Although freezing might be considered the riskier choice, most crayfish decide to stay near a food signal of high value rather than tail-flipping away from it to avoid a potential predator attack, thus balancing the different behavioral options based on a cost-benefit analysis. From Liden et al. (2010) (Figure 5A). (Reused with permission, Copyright 2010; The Royal Society)*.* **(B)**. Under standard food odor conditions, hungry (unfed) crayfish (*N* = 30) produce more freezes and fewer MG tail-flips than satiated (fed) crayfish (*N* = 29), indicating that intrinsic states affect their “economic” decision-making. From Schadegg and Herberholz (2017) (Figure 4). (Reused with permission, Copyright 2017; Springer-Verlag Berlin Heidelberg).

When juvenile crayfish were enticed to approach a food odor release point and suddenly exposed to a visual danger signal (i.e., shadow), they responded with only one of two incompatible behaviors, freezing or MG tail-flips (Liden and Herberholz 2008). The threshold for activating the MG neurons depended on both extrinsic and intrinsic conditions. For example, if the food quality was high, crayfish suppressed MG activation in response to the shadow and mostly froze (Figure 6A). This is certainly adaptive because a great meal is worth a bit more risk (Liden et al., 2010). If crayfish were hungry, the same happened for the same reason, the meal was of higher value and activation of MG tail-flips, which increased the distance to the food, was not the most desirable option (Schadegg and Herberholz 2017; Figure 6B). It is tempting to propose that these value-based (“neuroeconomic”) decisions in crayfish are mediated by TI acting on the MG threshold, but evidence for this is lacking. In fact, the neurons that produce TI in either of the two giant circuits have not been identified, and this is a missing piece critically needed for further advancement.

The notion of food- and hunger-related suppression of giant-mediated tail-flips was further supported by Kato et al. (2018). The authors compared habituation rates between fed or starved crayfish alone and in the presence of food after mechanical stimuli were directed to the rostral body parts. Although they did not measure whether the evoked tail-flip responses were in fact produced by the MG neurons or non-giant circuitry, they considered them “MG-type” tail-flips, and they found that starved crayfish habituated more quickly to the repeated stimuli with or without food present during the experiment. This implies that crayfish that are hungry or actively engaged with food (or its odor) lower the probability of costly escape by suppressing giant neuron excitability while also increasing its rate of habituation.

#### Neurochemical modulation

Numerous studies have investigated the effects of *biogenic amines* on crayfish neural circuits and behavior. It is impossible to cover all the literature here, and other than for the more recent developments, excellent reviews already exist (e.g., Krasne and Edwards 2002). In general, systemic injections of neuroactive substances into the hemocoel of freely behaving crayfish, or bath application (i.e., superfusion) of the crayfish isolated nervous system, in combination with electrophysiological and/or immunohistochemical approaches, have been productively used in many studies. The application methods mirror the naturally occurring neurohormonal actions that are produced when neurosecretory cells release circulating monoamines (and other substances such as neuropeptides) directly into the hemolymph, which reach their neuronal targets *via* the pericardial sinus, heart, and arteries that densely innervate the nervous tissue, including the ventral nerve cord (e.g., Brown and Sherwood 1981; Beltz 1999; Christie 2011).

Serotonin (5-HT), common to most nervous systems, has received most of the attention although there are examples for many others, including octopamine (OA). In 1980, Livingstone et al. (1980) reported that injections of 5-HT or OA into live crayfish (and lobster) elicited postural changes that resembled those seen in dominant or subordinate animals, respectively. This inspired a large number of subsequent studies, linking the actions of individual neuromodulators to social status in crayfish. Crayfish are highly aggressive animals and will determine social rank relationships during agonistic dyadic encounters from which one animal emerges as the dominant and the other one as the subordinate. During their fights for social dominance, crayfish tail-flip (a lot!) and employ a non-defensive fourth type of tail-flip (“offensive tail-flip”) to demonstrate physical fitness to their opponents (Herberholz et al., 2001). The threshold of the LG neurons changes during fights, but only in subordinates, which experience a substantial decrease in LG excitability (Krasne et al., 1997). Although this may seem counterintuitive at first since subordinates should have low threshold for escape when interacting with a fierce dominant opponent that keeps attacking them, the reason for this is similar to the idea discussed earlier for LG threshold changes during restraint. Suppression of LG excitability “frees up” shared circuit components that are used for more effective non-giant escape behavior. Another possibility, which also follows an argument used earlier, is that LG threshold in subordinates is high because crayfish typically face each other and interlock their claws at escalated fight intensities and being pushed into an obstacle by the stronger dominant opponent could trigger an unwanted forward-directed escape. However, none of these hypotheses has been explicitly tested.

How is LG threshold modulated? Based on earlier discussion, tonic inhibition might be at play. It seems reasonable to assume that a gradual increase in TI could change LG threshold in subordinates. This is speculative, however, and most of the existing evidence points towards 5-HT (and, albeit somewhat less investigated, OA).

Glanzman and Krasne (1983) found that LG excitability is affected by both amines, but in opposite directions. Using intracellular recordings of LG excitatory postsynaptic potentials (EPSPs) after stimulation of mechanosensory afferents, they demonstrated that the addition of 5-HT to the bathing solution of a dissected crayfish preparation produced a decrease of LG EPSP sizes, but an increase in EPSP sizes following superfusion with OA. The activity of Interneuron A, which provides excitatory input to LG, was also facilitated by OA, but unaffected by 5-HT. These experiments were done on socially isolated crayfish. Several years later, Yeh et al. (1996), Yeh et al. (1997) replicated the experiments (for 5-HT) in dominant and subordinate crayfish. They found that the effects of 5-HT were status-dependent, and 5-HT strongly reduced LG EPSPs in subordinates while causing an increase in dominants. This was a remarkable result because it showed that the effects of a single neurochemical on the same neuron depended on the social status of the animal. The authors also provided some explanation for the underlying mechanisms. Using different agonists for specific 5-HT receptor subtypes, they were able to show that the socially-mediated changes in LG excitability are likely related to differences in the expression of 5-HT_1_ and 5-HT_2_ receptors. In short, the overall take-away from this intriguing work is that 5-HT_1_ receptors suppress LG excitability while 5-HT_2_ receptors promote it, and thus, the LG of subordinate crayfish expresses more of the first type and the LG of dominants more of the second type. A next logical step would be to measure expression profiles of these receptors on the LG neurons, ideally comparing dominants and subordinates; however, this has not happened yet. Both receptor subtypes have been sequenced and cloned in crayfish (Spitzer et al., 2008) and given the recent developments in single-cell transcriptomics/proteomics, this appears to be a fruitful avenue for future studies (see below).

Yeh et al. (1996), Yeh et al. (1997) also measure 5-HT related LG EPSP changes in socially isolated crayfish. Contrary to earlier reports (Glanzman and Krasne 1983, 1986), they found that 5-HT increased LG EPSP size rather than reducing it. Eventually, researchers from both labs teamed up and discovered that the effects of 5-HT in isolates depend on the concentration of 5-HT and its application rate (Teshiba et al., 2001). For example, fast and short 5-HT application caused inhibition, which was opposite to facilitation observed after slow and long application. Thus, a single neuromodulator can have excitatory or inhibitory function on a single neuron depending not only on the social status of the animal, but also on its application parameters (Krasne and Edwards 2002). This further highlights the complex interrelationship between serotonin and nervous system function, suggesting that presynaptic release rate and postsynaptic receptor targets (and their corresponding second messengers) all influence the outcome.

Using isolated crayfish, Araki et al. (2005) investigated the effects of 5-HT and OA on habituation of the LG neurons. They found that both neuromodulators reduced habituation rates (i.e., more stimuli were needed to habituate LG), and they were able to link the effects of 5-HT to an increase of intracellular cAMP levels, while the effects of OA were independent of cAMP. The potentiation of LG responses to sensory stimulation by OA was later shown to depend on IP_3_ as the intracellular second messenger (Araki and Nagayama 2012). Thus, these two neuromodulators appear to activate separate signaling cascades when interacting with their respective LG receptors. The rate of LG habituation also varied with social status. Araki et al. (2013) and Nagayama and Araki (2015) found that the LG neurons of dominant and subordinate crayfish habituated more slowly when compared to isolated animals, and this effect lasted for at least 1 week after social status was established. These status-dependent changes of habituation were locally mediated and did not require brain-derived tonic inhibition.

**FIGURE 7:**
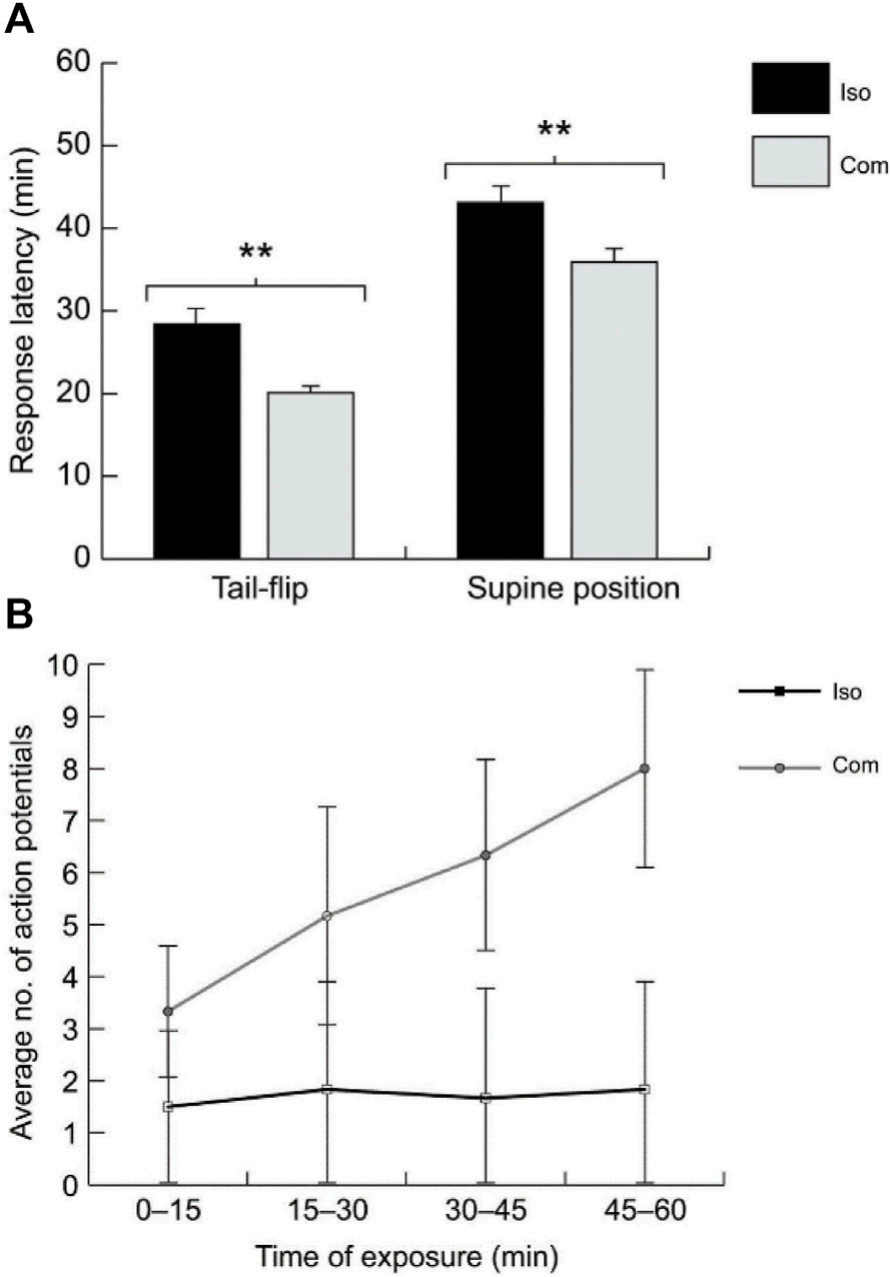
**(A)**. The behavioral latencies for spontaneous tail-flipping and supine posture (i.e., falling on the back) after acute alcohol (EtOH) exposure are significantly longer in isolated crayfish (ISO = 20) compared to communally housed animals (COM; *N* = 24). **(B)**. With increased exposure time to EtOH, the number of action potentials in the LG neuron evoked by constant stimulation of the sensory nerves increased in COMs (*N* = 7) compared to ISOs (*N* = 5). From Swierzbinski et al. (2017) (Figures 1, 4). (Reused with permission from The Company of Biologists).

Crayfish have also been shown to respond behaviorally and physiologically to several drugs of abuse, including cocaine, morphine, amphetamines, and ethanol (e.g., Friedman et al., 1988; Shipley et al., 2017). These effects have not been connected to the giant neurons, except for ethanol (EtOH). Swierzbinski et al. (2017) found that juvenile crayfish are behaviorally sensitive to EtOH, and this sensitivity is affected by the prior social experience of the animals (Figure 7). By placing crayfish into a mix of water and ethanol, they observed discrete behavioral changes as the animals became more intoxicated, from changes in posture (“elevated stance”) to spontaneous (unprovoked) tail-flipping and eventually loss of motor control (Figure 7A). The timing of these behavioral changes was found to be dose-dependent, i.e., crayfish progressed more quickly through all stages of intoxication at higher EtOH concentrations. Using reduced preparations, the authors further demonstrated that the LG neurons are sensitive to EtOH and LG EPSPs (and action potentials) elicited by constant sensory stimulation increased after EtOH exposure (Figure 7B). Together these results illustrated that crayfish taken from a communal tank exhibit higher behavioral and physiological sensitivity to EtOH compared to socially isolated crayfish.

Soon after, Swierzbinski and Herberholz (2018) showed that EtOH also facilitated the response of the MG neurons to sensory inputs. In addition, the authors highlighted the role of the GABAergic system in mediating this effect. Agonizing GABA_A_ receptors eliminated the EtOH facilitation of the MG neurons in socially isolated crayfish. The socially dependent effects of EtOH on the LG neurons in juvenile crayfish were later replicated in adults (Venuti et al., 2021). In addition, hemolymph EtOH concentration was found to be similar for communally housed and isolated crayfish, indicating that the social differences are not based on EtOH uptake or metabolism. The same study found that differences in the expression of GABA_A_ receptors are partially responsible for the lower EtOH sensitivity of the LG neurons in isolated crayfish. One type of GABA_A_ receptor pharmacologically identified in the study contained *delta* subunits, a receptor subtype found in other species, including mammals, which is located extrasynaptically and has high binding affinity for EtOH. Although still somewhat speculative, these results suggest that social isolation causes a change in the expression of specific receptors in the crayfish giant circuits, including those that are targeted by EtOH.

### The next decade

Although few neural circuits in the animal kingdom can rival the LG in terms of structural and functional understanding, there are still important gaps to fill in the coming years.

**FIGURE 8:**
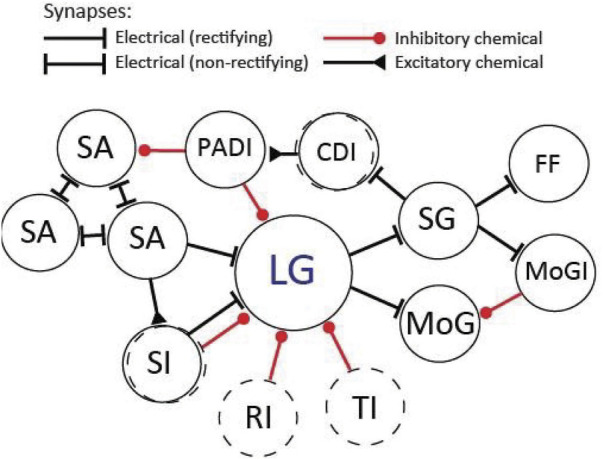
Diagram of the LG circuit as it may appear in the rostral abdominal ganglia (A1-A3). Not all connections are shown. The circuit primarily consists of (rectifying) electrical synapses. Excitation: On the sensory side LG receives electrical inputs from sensory afferents (SA) and sensory interneurons (SI). The sensory afferents couple electrically to each other *via* non-rectifying synapses. On the output side, LG makes electrical synapses with the motor giant (MoG) neurons and segmental giant (SG) neurons. The SGs make electrical synapses with the fast-flexor (FF) motor neurons, the motor giant inhibitor (MoGI) neurons, and the corollary discharge (CDI) interneurons. Inhibition: The LG receives inhibition from unidentified sensory interneurons (SI), from unidentified inhibitory neurons that produce recurrent inhibition (RI) as well as from unidentified descending interneurons that originate in the brain and produce tonic inhibition (TI). LG also receives direct inhibition from primary afferent depolarizing interneurons (PADI), which predominantly inhibit the terminals of SAs. Dashed circles indicate unidentified neuronal populations.

The LG circuit features an abundance of electrical neurotransmission (Figure 8). As mentioned earlier, the first (rectifying) electrical synapse was described by Furshpan and Potter between the presynaptic LG and postsynaptic MoG. Electrical transmission *via* gap junctions is abundant in the nervous systems of many animals, where it plays largely conserved and highly important functional roles (Connors and Long 2004; Pereda and Macagno 2017). Historically, however, electrical transmission has been overlooked in favor of chemical synapses, which were considered more adaptable and plastic. This has changed in recent years and new findings have uncovered surprising plasticity in electrical neurotransmission. For example, coupling across electrical synapses can be modulated by various mechanisms and on various timescales, and just like chemical synapses, gap junctions exhibit hallmarks of learning and memory such as long-term potentiation (LTP) and long-term depression (LTD.) (Curti and O’Brien 2016; Haas et al., 2016; Coulon and Landisman 2017). Importantly, prior work on the LG circuit has demonstrated LTP across the sensory input pathways, including the electrical synapses between interneurons and LG. Originally the potentiation of LG EPSPs after high-frequency stimulation of the sensory nerves that contain primary afferents was attributed to plasticity in the chemical synapses between afferents and interneurons (Miller et al., 1987), but this idea was later expanded to include the electrical synapses that connect the mechanosensory interneurons to LG (Tsai et al., 2005). In addition, the number of gap junctions changed across LG septate junctions after high-frequency mechanical stimulation of the tail, indicating plasticity in response to strong activation (Ohta et al., 2011). The LGs also form gap junctions on the output side with other large, identified neurons such as the MoGs and SGs (Mittenthal and Wine 1973; Leitch et al., 1989; Figure 8). Although invertebrate and vertebrate gap junction proteins differ in primary sequence, they are structurally similar (e.g., same membrane topology) and considered functionally identical (Phelan 2005; Skerrett and Williams 2017). Thus, the crayfish LG circuit is well suited to make important contributions to our limited understanding of electrical neurotransmission, including those of biomedical relevance since dysfunction of gap junctions has been implied in several major neurological diseases (e.g., Dere and Zlomuzica 2012; Aasen 2021).

**FIGURE 9:**
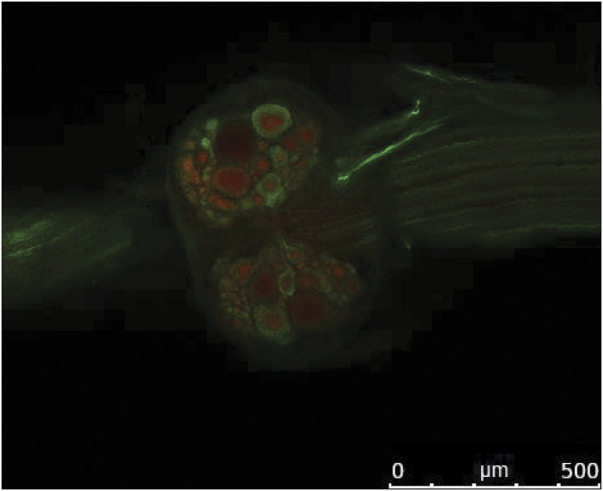
Confocal image illustrating immunolabeling of GABA (green) and GABA_A_ receptors (red) on the ventral side of the fifth abdominal ganglion of crayfish. Rostral is to the left. From Venuti Winter L.S. 2020; Ph.D. Thesis, ProQuest 27738217 (Reused with permission obtained from author).

Despite a large network of inhibition in the LG circuit, very few of the inhibitory neurons that participate in feedback, feedforward, recurrent, or tonic inhibition have been identified. This is somewhat surprising given the historical role of the crayfish model in discovering not only basic inhibitory mechanisms but providing first evidence of the inhibitory action of GABA, the main inhibitory neurotransmitter in most animal species (e.g., Hagiwara et al., 1960; Dudel 1962; Kravitz et al., 1963). What is relatively well known are the inhibitory output pathways that follow action potentials of the giant neurons. In fact, some of the largest cells in the abdominal ganglia, including those that mediate flexor and extensor inhibition of the tail muscles, often label prominently in immunocytochemical stains for GABA. Given the abundance of GABAergic cells and receptors found in crayfish ganglia (Figure 9), many of the early experiments on LG circuit inhibition measured the effects of picrotoxin, which blocks ligand-gated chloride channels. However, picrotoxin enters the pore of the channel rather than antagonizing the binding site of the ligand, and thus, part of the observed effects could also be mediated by glutamate-gated chloride channels, which will produce similar chloride-mediated inhibition in crayfish and other invertebrates (Heitler et al., 2001; Wolstenholme 2012). Since there is new urgency in understanding how the balance of excitation and inhibition affects homeostasis in healthy and diseased nervous systems (e.g., Sohal and Rubenstein 2019; Chen et al., 2022), the LG circuit of crayfish, which is rich in interconnected inhibitory and excitatory networks and provides access to single neurons that experience both events, seems well suited to productively contribute to this literature.

Undoubtedly, the LG circuit has provided enormous new insight into discrete neural mechanisms that regulate behavior. Analysis of the MG circuit, however, has progressed much slower, as already discussed earlier in this review. Additional work on the MG circuit is especially important given the behavioral relevance of MG tail-flips in both intra- and inter-specific interactions (Herberholz et al., 2001, Herberholz et al., 2004).

While this review is primarily focused on the giant circuits in crayfish and only makes occasional reference to the non-giant circuit and the behavior it controls, a better understanding of this circuit is of great interest as well. As mentioned before, non-giant tail-flips exhibit greater behavioral plasticity than giant-mediated tail-flips, are used more “voluntarily”, and allow for directional control and (related) improved escape rate after an attack. Unfortunately, our understanding of the non-giant circuitry is quite limited and fades in comparison to the LG and (even) the MG circuit. This is, at least in part, due to the challenges of mapping out a complex circuit without the luxury of having large, accessible neurons as starting points. Nevertheless, after Joan Schrameck (1970) first published the identification of a crayfish escape tail-flip without giant fiber activity, some exciting behavioral analyses and important work on the underlying mechanisms have been done, much of it provided by Jeff Wine and colleagues. This includes discoveries about different employment of non-giant tail-flips, either as a direct response to more gradually delivered “threat” stimuli, or as a sequence of repeated swimming movements, controlled by a central pattern generator, that sometimes follow a giant-mediated escape (e.g., Reichert et al., 1981; Reichert and Wine 1983; Kramer and Krasne 1984; Krasne and Wine 1984; Lee and Wine 1984; Figure 2). More research efforts directed to the structural and functional features of the non-giant circuit is certainly desirable, including its interactions with the giants in the context of decision-making during escape.

The giant circuits are highly suitable for a wide range of experimental approaches, but they clearly lag behind in productive experimental areas such as genomics and proteomics. Progress has been limited compared to other genetically tractable invertebrate models because fully sequenced and annotated genomic data for crayfish was not available until very recently (Gutekunst et al., 2018; Shi et al., 2018; Xu et al., 2021). Now, there seems to be a new urgency to integrate these useful molecular tools with existing experimental methods (Stein et al., 2022; see contribution in this special topics issue). This will certainly shift focus towards a new set of exciting experiments, including ones that feature the crayfish giant circuits, which provide years of existing, detailed knowledge about their physiological function, and which link directly to adaptive behavior.

Lastly, despite the wide abundance of different crayfish species (>350 in the US alone), comparative approaches on circuit mechanisms are rare. In fact, most research has been done in members of one genus, *Procambarus*, with the Red Swamp Crayfish (*Procambarus clarkii*) being the most popular species across research labs. Fewer, but equally important, contributions include other genera in the US as well as worldwide such as *Orconectes*, *Cherax*, *Astacus*, and *Pacifastacus*. All crayfish species have LG and MG giant escape circuits, which is not the case for other crustaceans (e.g., Espinoza et al., 2006), and they live in different habitats, face different predators, display varying levels of aggression, reproduce sexually or asexually, and so on. Thus, it would be interesting to better understand how much adaptation to different environments shapes circuit structure and function, a question that has been productively studied in other invertebrates such as mollusks (e.g., Orr et al., 2008; Newcomb et al., 2012; Sakurai and Katz 2017). Moreover, most crayfish labs perform their experiments on *wild-caught* crayfish, which provides another unique advantage for undertaking such ecologically relevant, comparative studies.

## Conclusion

Over the past century, research on the giant circuits of crayfish have generated significant knowledge on neural mechanisms that produce natural behavior. Much has been uncovered about circuit formation, structure, and function, but additional work is needed to fill existing gaps. The availability of new technologies (e.g., genetic tools) will aid in forming relevant questions and provide additional exciting opportunities for the future. Although not discussed here, the crayfish escape circuits have also provided unmatched training opportunities for students at all career levels, either in the lab or in the classroom. Despite the shift in funding priorities towards animal models of “higher” biomedical importance, the many advantages of the crayfish escape circuits for uncovering basic and generalizable, or equally important, *unique* neurobehavioral mechanisms continue to exist more than 100 years after their discovery.
